# Lowering LDL cholesterol reduces cardiovascular risk independently of presence of inflammation

**DOI:** 10.1016/j.kint.2017.09.011

**Published:** 2018-04

**Authors:** Benjamin C. Storey, Natalie Staplin, Richard Haynes, Christina Reith, Jonathan Emberson, William G. Herrington, David C. Wheeler, Robert Walker, Bengt Fellström, Christoph Wanner, Martin J. Landray, Colin Baigent, The SHARP Collaborative Group, Benjamin C. Storey, Natalie Staplin, Richard Haynes, Christina Reith, Jonathan Emberson, Will G. Herrington, David C. Wheeler, Robert Walker, Bengt Fellström, Christoph Wanner, Martin J. Landray, Colin Baigent, Colin Baigent, Martin J. Landray, Christina Reith, Jonathan Emberson, David C. Wheeler, Charles Tomson, Christoph Wanner, Vera Krane, Alan Cass, Jonathan Craig, Bruce Neal, Lixin Jiang, Lai Seong Hooi, Adeera Levin, Lawrence Agodoa, Mike Gaziano, Bertram Kasiske, Robert Walker, Ziad A. Massy, Bo Feldt-Rasmussen, Udom Krairittichai, Vuddidhej Ophascharoensuk, Bengt Fellström, Hallvard Holdaas, Vladimir Tesar, Andrzej Wiecek, Diederick Grobbee, Dick de Zeeuw, Carola Grönhagen-Riska, Tanaji Dasgupta, David Lewis, William Herrington, Marion Mafham, William Majoni, Karl Wallendszus, Richard Grimm, Terje Pedersen, Jonathan Tobert, Jane Armitage, Alex Baxter, Christopher Bray, Yiping Chen, Zhengming Chen, Michael Hill, Carol Knott, Sarah Parish, David Simpson, Peter Sleight, Alan Young, Rory Collins

**Affiliations:** 1 Medical Research Council Population Health Research Unit, Nuffield Department of Population Health, University of Oxford, Oxford, UK; 2 Clinical Trial Service Unit and Epidemiological Studies Unit, Nuffield Department of Population Health, University of Oxford, Oxford, UK; 3 Centre for Nephrology, University College London, London, UK; 4 Department of Medicine, University of Otago, Dunedin, New Zealand; 5 Renal Unit, Department of Medicine, University of Uppsala, Uppsala, Sweden; 6 Division of Nephrology, University Hospital, Würzburg, Germany; 7 Big Data Institute, Lia Ka Shing Centre for Health Information and Discovery, University of Oxford, Oxford, UK

**Keywords:** C-reactive protein, inflammation, LDL cholesterol, randomized trials, vascular disease

## Abstract

Markers of inflammation, including plasma C-reactive protein (CRP), are associated with an increased risk of cardiovascular disease, and it has been suggested that this association is causal. However, the relationship between inflammation and cardiovascular disease has not been extensively studied in patients with chronic kidney disease. To evaluate this, we used data from the Study of Heart and Renal Protection (SHARP) to assess associations between circulating CRP and LDL cholesterol levels and the risk of vascular and non-vascular outcomes. Major vascular events were defined as nonfatal myocardial infarction, cardiac death, stroke or arterial revascularization, with an expanded outcome of vascular events of any type. Higher baseline CRP was associated with an increased risk of major vascular events (hazard ratio per 3x increase 1.28; 95% confidence interval 1.19-1.38). Higher baseline LDL cholesterol was also associated with an increased risk of major vascular events (hazard ratio per 0.6 mmol/L higher LDL cholesterol; 1.14, 1.06-1.22). Higher baseline CRP was associated with an increased risk of a range of non-vascular events (1.16, 1.12-1.21), but there was a weak inverse association between baseline LDL cholesterol and non-vascular events (0.96, 0.92-0.99). The efficacy of lowering LDL cholesterol with simvastatin/ezetimibe on major vascular events, in the randomized comparison, was similar irrespective of CRP concentration at baseline. Thus, decisions to offer statin-based therapy to patients with chronic kidney disease should continue to be guided by their absolute risk of atherosclerotic events. Estimation of such risk may include plasma biomarkers of inflammation, but there is no evidence that the relative beneficial effects of reducing LDL cholesterol depends on plasma CRP concentration.

Inflammation has been implicated in the pathobiology of cardiovascular disease,^1^, ^2^ but it is unclear whether inflammation is a direct cause of disease or simply a marker of disease response. Among apparently healthy individuals, prospective cohort studies have shown that markers of inflammation, such as C-reactive protein (CRP)^3^ and interleukin-6^4^, ^5^ (IL-6), are positively associated with an increased risk of cardiovascular events, and studies employing Mendelian randomization have suggested that the association between IL-6 and risk may be causal.^6^ Prospective studies have also shown that low-density lipoprotein cholesterol (LDL-C) is positively associated with risk of major vascular events,^7^, ^8^ while randomized trials of statins^9^ (and, more recently, of proprotein convertase subtilisin/kexin type 9 or PCSK-9 inhibitors^10^) have shown that lowering LDL-C reduces cardiovascular risk, confirming that LDL-C is a cause of atherosclerotic disease. It is unclear whether the presence of inflammation influences the relationship between LDL-C and cardiovascular disease. Some groups have suggested, based on experimental studies, that the presence of inflammation may reduce the efficacy of statin therapy,^11^ but others have reported — albeit in nonrandomized analyses of randomized trials of statin therapy — that greater reductions in CRP with a statin are associated with larger reductions in cardiovascular risk.^12^, ^13^

Observational studies previously suggested that cholesterol might not be associated with increased cardiovascular risk in patients with chronic kidney disease (CKD).^14^, ^15^, ^16^ By contrast, the Study of Heart and Renal Protection (SHARP, which randomized 9270 patients with CKD to simvastatin 20 mg plus ezetimibe 10 mg daily or matching placebo), reported that during a median follow-up of 4.9 years, a mean reduction of 0.85 mmol/l LDL-C reduced the risk of major atherosclerotic events (a composite of nonfatal myocardial infarction [MI], coronary death, nonhemorrhagic stroke, and arterial revascularization) by 17%, (rate ratio 0.83, 95% confidence interval [CI] 0.74–0.94). Furthermore, the strength of the causal association was comparable to that observed in trials among people without CKD.^9^, ^17^ The most likely explanation for the discrepancy between observational studies and SHARP is that nonrandomized studies in patients with CKD are subject to “reverse causality,” whereby CKD (or comorbid disease) causes both lower LDL-C and an increased risk of death, thereby creating an apparent association between low cholesterol concentrations and death.^18^, ^19^

In patients with CKD, inflammation is independently associated with an increased risk of cardiovascular disease.^20^, ^21^, ^22^ It has been suggested that LDL-C may not be associated with cardiovascular disease among patients with evidence of inflammation. In one study of 823 incident dialysis patients, 77% of whom were defined as having evidence of inflammation (albumin < 36 g/l, CRP ≥10 mg/l, or IL-6 ≥3.09 pg/ml), non–high-density lipoprotein cholesterol (non-HDL-C, a close correlate of LDL-C) was not associated with cardiovascular mortality overall (hazard ratio [HR] per 1 mmol/l higher non-HDL-C 0.97; 95% CI 0.86–1.10), but there was a significant association (HR 2.09; 95% CI 1.02–4.27) among patients without evidence of inflammation.^23^

The SHARP trial provides a further cohort in which to explore the associations of inflammation and LDL-C, because cardiovascular (and nonvascular) outcomes were systematically recorded and measures of lipid profile and CRP were taken at baseline and (in a subset of patients) at follow-up. This affords the opportunity to examine: (i) the association of CRP with risk of cardiovascular disease; (ii) the associations, in randomized and observational analyses, of LDL-C with risk of cardiovascular disease; (iii) whether the degree of underlying inflammation modified the strength of the association between LDL-C and cardiovascular risk in this population; and (iv) the separate associations of CRP and of LDL-C with nonvascular outcomes.

## Results

Among 9270 patients randomized to simvastatin/ezetimibe versus placebo, 8603 had a plasma CRP concentration measured at baseline. Compared with patients with baseline CRP <3 mg/l, patients with CRP ≥3mg/l were older (63 vs. 61 years), more likely to be male, (64% vs. 61%), had a higher body mass index (28.3 vs. 25.8 kg/m^2^), had more prior vascular disease (17% vs. 13%), and were more likely to be on dialysis (39% vs. 26%). Mean LDL-C was slightly lower among those with higher CRP (2.74 vs. 2.82 mmol/l) (Table 1).

**Table 1:** Baseline characteristics by C-reactive protein group among all 9270 SHARP participants

Baseline characteristic	C-reactive protein (mg/l)		
	<3 (*n* = 4298)	≥3 (*n* = 4305)	Not available (*n* = 667)
CRP (mg/l) (median, IQR)	1.2 (0.7–2.0)	7.1 (4.6–13.5)	-
Age at randomization (yrs)	61 (12)	63 (12)	62 (12)
Men	2630 (61%)	2773 (64%)	397 (60%)
Prior vascular disease	576 (13%)	733 (17%)	84 (13%)
Diabetes	917 (21%)	1034 (24%)	143 (21%)
Current smoker	532 (12%)	615 (14%)	87 (13%)
Diastolic blood pressure (mm Hg)	80 (12)	78 (13)	78 (13)
Systolic blood pressure (mm Hg)	139 (21)	139 (23)	140 (22)
LDL cholesterol (mmol/l)	2.82 (0.89)	2.74 (0.86)	2.70 (0.85)
HDL cholesterol (mmol/l)	1.17 (0.35)	1.06 (0.32)	1.10 (0.26)
Apolipoprotein A1 (mg/dl)	139 (29)	128 (28)	136 (30)
Apolipoprotein B (mg/dl)	96 (26)	97 (25)	94 (25)
Albumin (g/l)	40.7 (3.6)	39.5 (3.7)	-
Body mass index (kg/m^2^)	25.8 (4.5)	28.3 (6.1)	27.3 (6.0)
Ethnicity			
White	2944 (68%)	3258 (76%)	444 (67%)
Black	88 (2%)	120 (3%)	56 (8%)
Asian	1150 (27%)	803 (19%)	133 (20%)
Other/not specified	116 (3%)	124 (3%)	34 (5%)
Co-medication			
Antiplatelet therapy	852 (20%)	1112 (26%)	141 (21%)
ACE inhibitor or ARB	2447 (57%)	2263 (53%)	320 (48%)
Beta blocker	1620 (38%)	1657 (38%)	237 (36%)
Calcium channel blocker	1891 (44%)	1694 (39%)	255 (38%)
Renal status			
Not on dialysis	3188 (74%)	2631 (61%)	426 (64%)
On dialysis	1110 (26%)	1674 (39%)	241 (36%)
MDRD-estimated GFR (ml/min per 1.73m^2^)^a^^,^^b^			
Mean (SD)	26.9 (13.7)	26.2 (12.2)	25.4 (12.4)
≥60	61 (2%)	26 (1%)	1 (0%)
≥30 to <60	1159 (36%)	924 (35%)	72 (34%)
≥15 to <30	1315 (41%)	1162 (44%)	88 (42%)
<15	652 (20%)	518 (20%)	49 (23%)
Not available	1	1	216
Urinary albumin:creatinine ratio (mg/g)^a^^,^^b^			
Median (IQR)	206 (50–725)	206 (51–645)	206 (206–206)
<30	584 (20%)	481 (20%)	42 (22%)
≥30 to ≤300	1120 (38%)	925 (39%)	63 (32%)
>300	1280 (43%)	987 (41%)	90 (46%)
Not available	204	238	231

ACE, angiotensin-converting enzyme; ARB, angiotensin II receptor blocker; CRP, C-reactive protein; GFR, glomerular filtration rate; HDL, high-density lipoprotein; IQR, interquartile range; LDL, low-density lipoprotein; MDRD, Modification of Diet in Renal Disease Study.

Data are *n* (%), mean (SD), or median (IQR).

a Among patients not on dialysis.

b Percentages exclude participants for whom data were not available for that category.

During a median follow-up of 4.9 years, 2317 patients experienced at least 1 vascular event of any type; 1406 such events were adjudicated to be atherosclerotic and 1342 were nonatherosclerotic. Of 2317 vascular events of any type, 1515 met the definition of major vascular events.

Among 962 patients with measurements of CRP and LDL-C at both baseline and 2.5 years, allocation to simvastatin/ezetimibe produced an average 0.99 mmol/l (SE 0.06 mmol/l) reduction in LDL-C (or a 35% [SE 2.0] proportional reduction) with similar absolute (and proportional) reductions irrespective of baseline CRP (Table 2). Allocation to simvastatin/ezetimibe produced an average 0.23 (SE 0.08) log mg/l reduction in log CRP, which equated to a 21% (SE 7.1) proportional reduction. In terms of the effect size relative to the SD of baseline measurements, allocation to simvastatin/ezetimibe had a greater effect on LDL-C (1.1 SD difference) than log CRP (0.2 SD difference).

**Table 2:** Effect of allocation to simvastatin plus ezetimibe on changes in concentrations of LDL cholesterol and CRP between baseline and study midpoint (2.5 yr)

Change in CRP or LDL-C	*n*	Mean (SD) of baseline values	Change in mean concentration (SE) from baseline to 2.5 years			Difference as proportion of baseline mean (SE)	Difference in number of SDs of baseline values (SE)
			Simvastatin plus ezetimibe	Placebo	Absolute difference		
*Changes in log CRP (log mg/l)*							
All patients	962	1.1 (1.3)	–0.11 (0.06)	0.12 (0.05)	–0.23 (0.08)	–21% (7.1)	–0.2 (0.06)
Baseline LDL-C (mmol/l)							
<2.72	464	1.1 (1.4)	–0.12 (0.08)	0.09 (0.08)	–0.21 (0.11)	–19% (10.2)	–0.2 (0.08)
≥2.72	498	1.1 (1.3)	–0.10 (0.08)	0.15 (0.07)	–0.25 (0.11)	–23% (9.9)	–0.2 (0.08)
Baseline CRP (mg/l)							
<3	473	0.03 (0.73)	0.25 (0.07)	0.51 (0.07)	–0.26 (0.10)	–804% (308.9)	–0.4 (0.14)
≥3	489	2.13 (0.83)	–0.49 (0.08)	–0.24 (0.07)	–0.25 (0.11)	–12% (5.1)	–0.3 (0.13)
*Changes in LDL-C (mmol/l)*							
All patients	962	2.79 (0.88)	–1.19 (0.04)	–0.21 (0.04)	–0.99 (0.06)	–35% (2.0)	–1.1 (0.06)
Baseline LDL-C (mmol/l)							
<2.72	464	2.1 (0.4)	–0.77 (0.05)	0.13 (0.04)	–0.90 (0.06)	–43% (3.0)	–2.0 (0.14)
≥2.72	498	3.4 (0.7)	–1.57 (0.06)	–0.53 (0.05)	–1.04 (0.08)	–30% (2.3)	–1.5 (0.11)
Baseline CRP (mg/l)							
<3	473	2.84 (0.94)	–1.22 (0.06)	–0.32 (0.05)	–0.90 (0.08)	–32% (2.7)	–1.0 (0.08)
≥3	489	2.74 (0.82)	–1.17 (0.06)	–0.10 (0.05)	–1.07 (0.08)	–39% (3.0)	–1.3 (0.10)

CRP, C-reactive protein; LDL, low-density lipoprotein; LDL-C, LDL cholesterol.

Only patients with samples of both LDL cholesterol and CRP at baseline and 2.5 years were included in these analyses. Average compliance to allocated treatment was about 70% irrespective of baseline LDL-C or CRP.

### Association between C-reactive protein and vascular events

**Figure 1:**
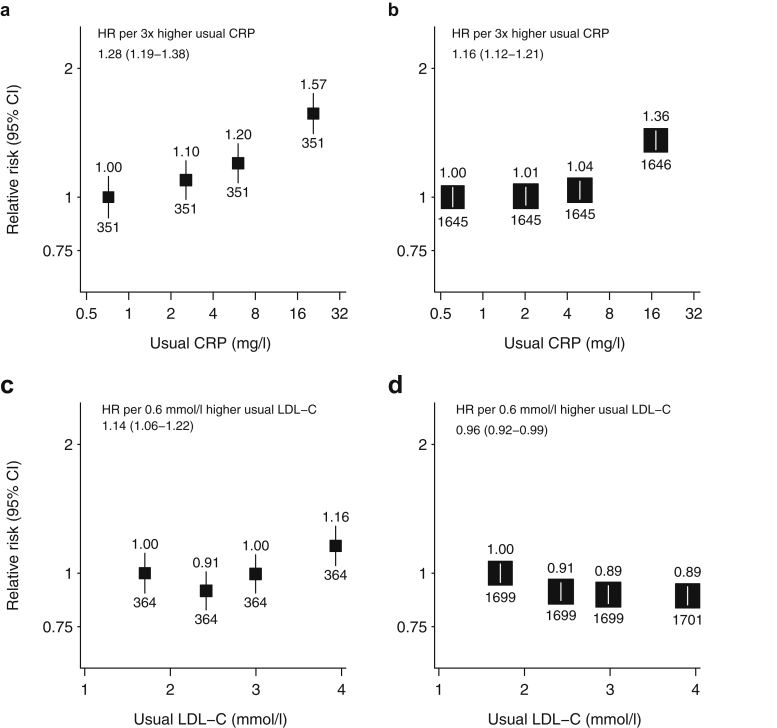
**Association between usual C-reactive protein (CRP) and the risk of (a) major vascular events and (b) nonvascular events, and the association between usual low-density lipoprotein cholesterol (LDL-C) and the risk of (c) major vascular events and (d) nonvascular events.** Hazard ratios (HRs) adjusted for age, sex, ethnicity, treatment allocation, prior diabetes, prior vascular disease, smoking, body mass index, high-density lipoprotein cholesterol, and renal status are noted (above squares), along with numbers of events (below squares). Average HR (95% confidence interval [CI]) throughout the range of values studied (i.e., assuming a log-linear relationship for LDL−C and a log-log-linear relationship for CRP), corresponding to about 1 SD difference in the usual LDL−C/log CRP.

Each 3-fold increase in usual CRP (or, equivalently, 1 SD higher usual log CRP) was associated with 28% higher major vascular event (MVE) risk (HR 1.28, 1.19–1.38; Figure 1a), and was similar irrespective of baseline LDL-C (HRs 1.39 [1.24–1.56] if LDL-C <2.72 mmol/l vs. 1.22 [1.10–1.35] if LDL-C ≥2.72 mmol/l, *P* for interaction = 0.07, Supplementary Table S1). The association between usual CRP and vascular events of any type was of similar magnitude (1.28 [1.20–1.36]; Supplementary Figure S2B), and this association was similar for both atherosclerotic (1.21, 1.12–1.31) and nonatherosclerotic vascular events (1.34, 1.23–1.45; Supplementary Figures S2C and S2D; *P* for difference = 0.09).

### Association between LDL-cholesterol and vascular events

Among the 9270 randomized patients, allocation to simvastatin/ezetimibe reduced the risk of MVE by 15% (risk ratio [RR] 0.85, 0.77–0.94, *P* = 0.0012). In nonrandomized (observational) analyses; however, usual LDL-C was only weakly associated with risk of MVE, with each 0.6 mmol/l increase in (or 1 SD higher) LDL-C associated with a 14% increased risk of MVE (HR 1.14, 95% CI 1.06–1.22; Figure 1c and Supplementary Figure S3A). The association between usual LDL-C and vascular events of any type was also weakly positive (HR per 0.6 mmol/l 1.06, 1.00–1.12; Supplementary Figure S3B), reflecting a positive association with atherosclerotic vascular events (HR per 0.6 mmol/l 1.19, 1.11–1.28) but no significant association with nonatherosclerotic vascular events (HRs per 0.6 mmol/l 0.90, 0.83–0.97; Supplementary Figure S3C and S3D; *P* for difference < 0.0001).

### Influence of CRP on association between LDL-C and vascular events

**Figure 2:**
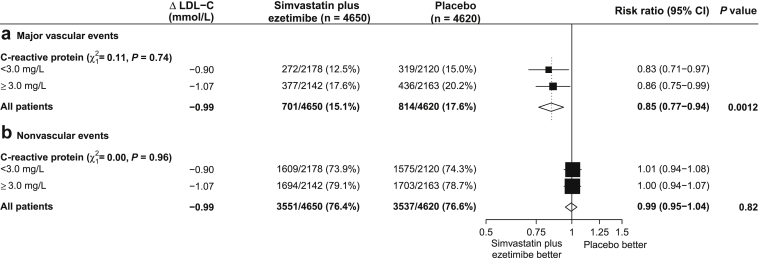
**Effect of allocation to simvastatin plus ezetimibe on (a) major vascular events and (b) nonvascular events by level of C-reactive protein.** CI, confidence interval; LDL-C, low-density lipoprotein cholesterol.

In the randomized comparison of simvastatin/ezetimibe versus placebo, the proportional risk reduction was similar in those with CRP <3 mg/l (RR 0.83, 0.71–0.97) and CRP ≥3 mg/l (RR 0.86, 0.75–0.99; *P* for interaction = 0.74; Figure 2a). The interactions between baseline CRP and risk reduction were also nonsignificant in the nondialysis and dialysis subgroups separately (Supplementary Figure S4). In the observational analyses, the association was also similar irrespective of baseline CRP (HRs for ;<3 mg/l vs. ≥3 mg/l: 1.15, 1.03–1.27 vs. 1.18, 1.08–1.30; *P* for heterogeneity = 0.67, Supplementary Table S1).

### Associations between CRP or LDL-C and nonvascular outcomes

Each 1 SD increase in usual CRP was associated with a 16% higher risk of any nonvascular outcome (HR 1.16, 1.12–1.21; Figure 1b). The strongest association was with respiratory events (HR 1.37, 1.27–1.48), but all types of nonvascular event had a statistically significant positive association with usual CRP (Supplementary Figure S5).

By contrast, allocation to simvastatin/ezetimibe had no effect on the risk of nonvascular events (RR 0.99, 0.95–1.04, *P* = 0.82), regardless of baseline CRP (*P* for interaction = 0.96; Figure 2). In observational analyses, there was a weak inverse association between usual LDL-C and the composite of all nonvascular outcomes (HR per 0.6 mmol/l 0.96, 0.92–0.99; Figure 1d) and this association appeared qualitatively similar for each of the disease categories studied (Supplementary Figure S6).

## Discussion

This study provides several insights into the inter-relationships between inflammation (as measured by CRP), LDL-C, and cardiovascular disease in a population with moderate-to-severe CKD. CRP has previously been shown to be positively associated with all-cause mortality in studies among patients with CKD,^24^, ^25^ but its association with vascular events has been uncertain.^21^, ^24^ This study shows clearly that CRP is associated with both atherosclerotic and nonatherosclerotic vascular disease, as well as many nonvascular diseases.

Although our study cannot directly address the question of whether inflammation causes cardiovascular disease, its results do not provide any support for this hypothesis in patients with CKD. CRP was positively associated with both atherosclerotic and nonatherosclerotic vascular events. Furthermore, positive associations were observed between CRP and a wide range of nonvascular outcomes in different body systems. Although counter-examples exist (e.g., cigarette smoking causes a range of vascular and nonvascular conditions),^26^ this lack of specificity is in contrast to the specificity of the association of LDL-C with atherosclerotic vascular events in this study and other randomized trials of statin therapy. As originally proposed by Hill,^27^ a nonspecific association of an exposure with many unrelated outcomes is evidence against a causal association.

The previously reported main results of the randomized comparison in SHARP demonstrated that LDL-C is a cause of atherosclerotic disease in people with CKD, and that the strength of the association is comparable to that observed in people without CKD.^17^ This suggests that previous observational studies in which the association between cholesterol and cardiovascular disease was weak^28^ or absent^14^, ^15^, ^16^ may have been subject to bias and confounding. In the present observational analyses of SHARP, the relationship between LDL-C and cardiovascular disease was weaker than would be expected. The results of our study reinforce the importance of careful phenotyping of cardiovascular events when exploring associations between a biomarker and such outcomes in patients with CKD, because there was clear evidence that the association between usual LDL-C and atherosclerotic vascular events was positive (as expected), whereas the association with nonatherosclerotic vascular events (e.g., heart failure and arrhythmias) was inverse. This suggests that studies that do not discriminate between vascular events of different etiology, as for example when examining associations with “cardiovascular mortality,” may be misleading.^15^, ^23^

Our study highlights the potential dangers of relying on observational studies to determine the role of inflammation in determining the effects of lowering LDL-C in this population. An analysis of an observational study, for example, was the basis for a previous claim that cholesterol may not be associated with cardiovascular risk in dialysis patients in whom there is evidence of inflammation.^23^ The present randomized study shows clearly that reducing LDL-C is effective irrespective of inflammatory status, as measured by baseline CRP. This is consistent with previous analyses of the Heart Protection Study (the largest statin trial which included patients with a broad range of baseline CRP values) and in other statin trials, including 2 conducted in hemodialysis patients, all of which found no evidence that the proportional effects of statin therapy differed according to baseline CRP.^29^, ^30^, ^31^, ^32^

Whereas inflammation is a feature of atherosclerosis, it remains unclear whether it is a cause of disease. Mendelian randomization (MR) studies in people without CKD indicate that genetic variants associated with higher CRP concentrations are not associated with higher vascular risk; that is, the CRP molecule itself is not a cause of atherosclerosis.^33^ MR studies of more proximal biomarkers of inflammation (e.g., IL-6) do, however, suggest a causal relationship between IL-6 and vascular disease.^6^ This hypothesis may be supported by the recent results of a trial of the anti-inflammatory agent canakinumab (an IL-1β antagonist), which has reported a positive result.^34^

It has previously been claimed that statins are most effective in the presence of inflammation and that patients achieving the greatest CRP reductions due to statin therapy experience the largest risk reductions, which would be consistent with a causal role for inflammation in atherosclerosis.^13^ This type of analysis is subject to bias, however, because the types of patients in whom larger reductions in CRP are achieved differ in other ways from those achieving smaller reductions, and adjustment for such differences may not be possible using recorded variables. Unbiased assessment of the role of the magnitude of reductions in CRP (as a marker for the degree of attenuation of the inflammatory response) would require the magnitude of change in CRP to be measured among all patients *before* randomization (e.g., during a “run-in” period on active drug). Alternatively, evidence of a larger effect among those with higher baseline CRP (or other indices of inflammation) may emerge from randomized controlled trials of anti-inflammatory therapies that are currently testing whether reducing inflammation can reduce vascular risk,^36^ although this was not observed in a recent trial of anti-IL-1ß therapy.^34^, ^35^

Our analyses had a number of limitations. Analyses of the SHARP trial as an observational study may be subject to collider bias when exploring associations between baseline measures and outcomes, because patients were highly selected (e.g., for the presence of CKD).^37^ A further limitation was that CRP was not measured in all patients during follow-up, so we were unable to determine the change in CRP among patients with different baseline levels of CRP. The strengths of the study include the detailed phenotyping of patients at baseline and adjudication of events occurring after randomization, and the central laboratory measurement of LDL-C and CRP, with repeat measures allowing correction for regression dilution.^38^ Most importantly, the trial provided an unbiased test of the etiological role of LDL-C among patients with different levels of inflammation through randomization to simvastatin/ezetimibe versus placebo.

In conclusion, our results show that both LDL-C and CRP are positively associated with the risk of MVE among patients with CKD, and that the association between LDL-C and cardiovascular disease is not significantly dependent on the severity of any underlying inflammatory response. The current recommendation that LDL-C–lowering therapy is offered to patients with CKD based chiefly on their absolute risk remains valid,^39^ and although this risk may be estimated with a risk score that includes data on inflammatory response, indices of inflammation in isolation should not influence such decisions.

## Methods

### Study design and participants

Details of the SHARP trial objectives, design, and methods have been reported previously.^40^ In brief, SHARP was an international, prospective, randomized controlled trial of people aged 40 years and older. Eligible participants had more than 1 previous measurement of serum or plasma creatinine of at least 150 μmol/l (1.7 mg/dl) in men or 130 μmol/l (1.5 mg/dl) in women, whether they were receiving dialysis or not. Participants with prior MI or coronary revascularization were excluded. Potentially eligible participants attended a screening visit at which a medical and drug history and anthropometric and blood pressure measurements were recorded and informed consent obtained. The study is registered at ClinicalTrials.gov (NCT00125593 and ISRCTN54137607). Ethics approval was obtained at all study sites before enrollment.

After 6 weeks of placebo run-in, eligible participants were randomized at a ratio of 4:4:1 to simvastatin 20 mg plus ezetimibe 10 mg (as a single tablet), placebo, or simvastatin 20 mg alone, with treatment allocation masked using a double-dummy method. After 1 year, those patients allocated to simvastatin alone who were alive and willing to continue were re-randomized to simvastatin/ezetimibe or placebo.

### Study procedures

Scheduled clinic visits were at 2 and 6 months after randomization and thereafter at 6 monthly intervals until the study end. Information was recorded at each follow-up about any suspected MI, stroke, vascular procedure, cancer, other reasons for hospital admissions, or other serious adverse events (SAEs). If a participant became unwilling or unable to attend follow-up visits, information about SAEs was obtained from them (or their relative or carer) by telephone or from their physicians until the scheduled end of the study. Local study staff then sought extra information from hospital records and other appropriate sources about all reports of SAEs that might relate to the trial’s major outcomes (MVE: a composite outcome consisting of nonfatal MI, cardiac death, nonfatal or fatal stroke, and arterial revascularization, excluding dialysis access procedures; end-stage renal disease; cancer; and death). This information was sent to the international coordinating center for central adjudication, in accordance with pre-specified definitions, by trained clinicians who were masked to study treatment allocation. For the current report, the main analyses are of MVEs and nonvascular events (subdivided into cancer, renal, respiratory, hepatobiliary or gastrointestinal, and other medical or trauma SAEs).^41^ In order to investigate the association between CRP, LDL-C, and different types of vascular disease further, we specified an expanded outcome of “vascular events of any type,” defined as atherosclerotic events (nonfatal MI, coronary death, arterial revascularization, unstable angina, heart failure due to coronary disease, ischemic stroke, transient ischemic attack, aortic aneurysm, limb ischemia, embolism, or thrombosis) and nonatherosclerotic vascular events (noncoronary cardiac death, heart failure not related to ischemic heart disease, arrhythmias, valvular heart disease, pericardial disease, hemorrhagic stroke, or subarachnoid hemorrhage) (Supplementary Appendix).

Blood and urine samples were collected from all participants at baseline and 2.5 years after randomization. In addition, for a random sample of 10% of participants, further blood samples were collected at 1 and 4 years after randomization. LDL-C was measured on a Beckman Coulter (Brea, CA) LX20/DxC 800 in all participants at all times, with a maximum coefficient of variation (CV) of 2.71%. CRP was measured on a Siemens (Washington, DC) BN ProSpec system with a maximum CV of 3.11%. CRP was measured in all participants at baseline and in a randomly selected 10% of participants at 2.5 years.

### Statistical analyses

For observational analyses, the associations between “usual” (i.e., long-term average) CRP or LDL-C and MVE were estimated using Cox proportional hazards regression, with the proportional hazard assumption tested through examination of the time-dependency of the Schoenfeld partial residuals. Regression dilution ratios were calculated from the 2.5-year repeat measurements of LDL-C and CRP (which only requires repeat measurements in a subset of participants) and a standard correction applied to the corresponding HRs.^38^ Based on assumptions about causal relationships between measured characteristics and outcomes^42^ (see directed acyclic graph; Supplementary Figure S1), analyses were adjusted for age, sex, ethnicity (white, black, Asian, or other), treatment allocation, prior disease (including diabetes mellitus and vascular disease), smoking status (current, former, or never), body mass index, HDL cholesterol, and renal status (estimated glomerular filtration rate [eGFR] ≥30 ml/min per 1.73m^2^; eGFR ≥15 to <30 ml/min per 1.73m^2^; eGFR <15 ml/min per 1.73m^2^; or on dialysis). Participants with missing values for CRP or LDL-C were excluded from the relevant analyses.

In figures, participants are grouped into 4 categories containing similar numbers of events, with the HR for each group plotted against the mean CRP or LDL-C concentration at the study midpoint and accompanied by a CI derived only from the variance of the log risk in that single group. Hence, each HR, including that for the reference group, is associated with a group-specific CI that can be thought of as reflecting the amount of data only in that 1 group, and allowing appropriate statistical comparisons to be made between any 2 groups.^43^

We conducted (*post hoc*) intention-to-treat analyses of the effect of allocation to simvastatin/ezetimibe on time to first MVE among patients below or above the median CRP (<3 mg/l, ≥3 mg/l). Standard log-rank methods were used to provide estimates of average event rate ratios, associated 95% CIs, and 2-sided *P* values. Standard chi-square tests for heterogeneity were used to compare event rate ratios between subgroups defined by baseline CRP. *P* values <0.05 were deemed to be conventionally statistically significant, but when interpreting the clinical relevance of findings, the *post hoc* nature of the analyses, the number of events available, and the magnitude of the *P* value were all considered.

## Disclosure

SHARP was funded by Merck/Schering-Plough Pharmaceuticals (North Wales, PA), with additional support from the Australian National Health and Medical Research Council, the British Heart Foundation, and the UK Medical Research Council. SHARP was initiated, conducted, and interpreted independently of the principal study funder (Merck & Co. and Schering Plough Corp., which merged in 2009). The Clinical Trial Service Unit of the University of Oxford (Oxford, UK) has a staff policy of not accepting honoraria or other payments from the pharmaceutical industry, except for the reimbursement of costs to participate in scientific meetings. RH, DCW, CW, MJL, and CB report other grants for unrelated work. In addition, DCW and CW have received consultancy lecture fees for unrelated work.​ All the other authors declared no competing interests.

## Supplementary Material

Supplementary Appendix S1 Definition of vascular events of any type, subdivided into atherosclerotic and nonatherosclerotic vascular events.

Table S1 Association between C-reactive protein (CRP) and LDL cholesterol (LDL-C) and major vascular events at high and low levels of LDL-C or CRP.

Figure S1 Causal diagram showing the assumed associations between baseline C-reactive protein, LDL cholesterol, vascular events, and other characteristics.

Figure S2 Association between usual C−reactive protein and the risk of (A) major vascular events, (B) vascular events of any type, (C) atherosclerotic vascular events, and (D) nonatherosclerotic vascular events.

Figure S3 Association between usual LDL cholesterol and the risk of (A) major vascular events, (B) vascular events of any type, (C) atherosclerotic vascular events, and (D) nonatherosclerotic vascular events.

Figure S4 Effect of allocation to simvastatin plus ezetimibe on major vascular events, by level of C−reactive protein and dialysis status.

Figure S5 Association between C−reactive protein and the risk of nonvascular events.

Figure S6 Association between LDL cholesterol and the risk of nonvascular events.
