# Supplementary material for: Lowering LDL cholesterol reduces cardiovascular risk independently of presence of inflammation
Source: Kidney Int. 2018 Apr;93(4):1000–7. doi: 10.1016/j.kint.2017.09.011 (PMC5978933; doi:10.1016/j.kint.2017.09.011)
Supplement: Supplementary Appendix S1 — Definition of vascular events of any type, subdivided into atherosclerotic and nonatherosclerotic vascular events. [file mmc1.pdf]

Supplementary Appendix: Definition of vascular events of any type, subdivided into atherosclerotic and non-atherosclerotic vascular events

Vascular events of any type

- *Atherosclerotic vascular event*
  - Major coronary events: non-fatal myocardial infarction or coronary death
  - Ischaemic stroke
  - Arterial revascularization: coronary or non-coronary (excluding interventions on haemodialysis access)
  - Other atherosclerotic coronary events: unstable angina, heart failure related to ischaemic heart disease
  - Other atherosclerotic cerebrovascular events: transient ischaemic attack, amaurosis fugax, retinal artery occlusion
  - Other atherosclerotic peripheral arterial disease:
    - aortic aneurysm (including rupture and dissection)
    - limb ischaemia, limb artery embolism or thrombosis
- *Non-atherosclerotic vascular event*
  - Non-coronary cardiac death
  - Heart failure not related to ischaemic heart disease (hypertrophic cardiomyopathy, cor pulmonale)
  - Arrhythmias: atrial fibrillation, ventricular tachycardia, other tachycardia, bradycardia/heart block, other arrhythmia, cardiac arrest
  - Valvular heart disease (including valve repair or replacement)
  - Pericardial disease: pericarditis, effusion
  - Haemorrhagic stroke
  - Subarachnoid haemorrhage
