# Supplementary material for: Lowering LDL cholesterol reduces cardiovascular risk independently of presence of inflammation
Source: Kidney Int. 2018 Apr;93(4):1000–7. doi: 10.1016/j.kint.2017.09.011 (PMC5978933; doi:10.1016/j.kint.2017.09.011)
Supplement: Table S1 — Association between C-reactive protein (CRP) and LDL cholesterol (LDL-C) and major vascular events at high and low levels of LDL-C or CRP. [file mmc2.pdf]

## Supplementary Table S1: Association between C-reactive protein and LDL-cholesterol and major vascular events at high and low levels of LDL-C/CRP

| | HR (95% CI) |
|---|---|
| CRP, per 3x higher usual level* (adjusted for LDL-C) | |
| Low LDL-C | 1.22 (1.10-1.35) |
| High LDL-C | 1.39 (1.24-1.56) |
| Overall | 1.29 (1.19-1.39) |
| LDL-C, per 0.6 mmol/L higher usual level† (adjusted for CRP) | |
| Low CRP | 1.15 (1.03-1.27) |
| High CRP | 1.18 (1.08-1.30) |
| Overall | 1.17 (1.09-1.25) |

CRP=C-reactive protein. LDL-C=LDL-cholesterol. HR=hazard ratio. CI=confidence interval. All analyses adjusted for age, sex, ethnicity, treatment allocation, prior diabetes, prior vascular disease, smoking, BMI, HDL cholesterol and renal status. *Average HR per 3x higher usual CRP across range of values studied (i.e. assuming a log-log-linear relationship). Tests for differences between slopes in LDL-C subgroups: $\chi^2=3.25$; p=0.07. †Average HR per 0.6 mmol/L higher usual LDL-C across range of values studied (i.e. assuming a log-linear relationship). Tests for differences between slopes in CRP subgroups: $\chi^2=0.19$; p=0.67.
