# Supplementary material for: Lowering LDL cholesterol reduces cardiovascular risk independently of presence of inflammation
Source: Kidney Int. 2018 Apr;93(4):1000–7. doi: 10.1016/j.kint.2017.09.011 (PMC5978933; doi:10.1016/j.kint.2017.09.011)
Supplement: Figure S1 — Causal diagram showing the assumed associations between baseline C-reactive protein, LDL cholesterol, vascular events, and other characteristics. [file mmc3.pdf]

Supplementary Figure S1: Causal diagram showing the assumed associations between baseline C-reactive protein, LDL-cholesterol, vascular events and other characteristics

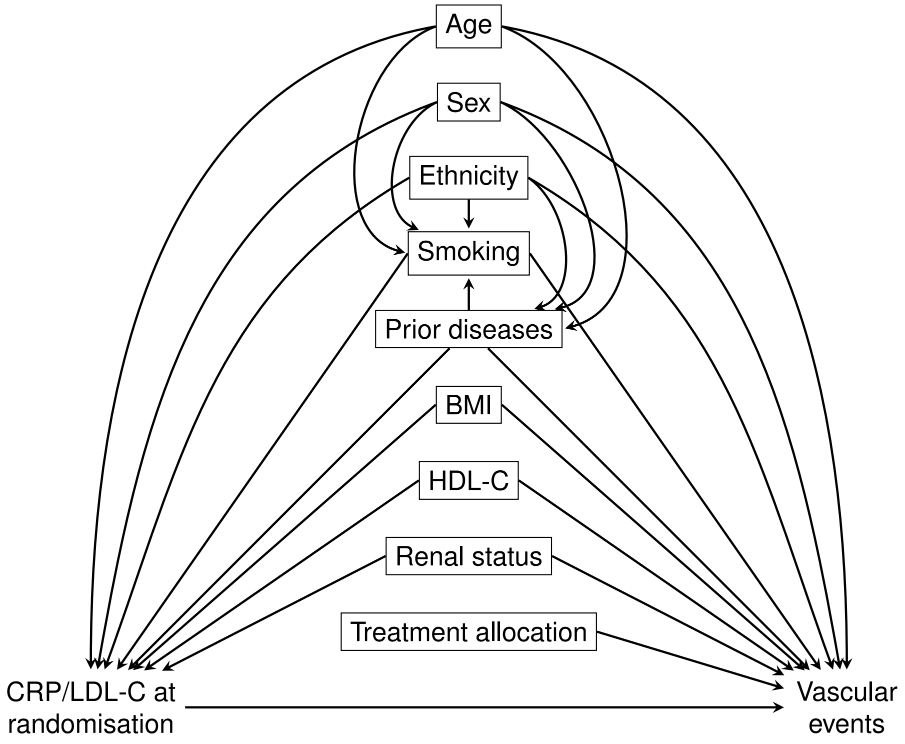

CRP = C-reactive protein. LDL-C = LDL cholesterol. BMI = body mass index. HDL-C = HDL cholesterol.
The arrows in the causal diagram represent the assumed directions of the associations between the characteristics. Any characteristics enclosed in boxes were included as covariates in the regression models used to estimate the observational associations between CRP/LDL-C and vascular events. See Reference 41 for further details on causal diagrams.
