# Supplementary material for: Lowering LDL cholesterol reduces cardiovascular risk independently of presence of inflammation
Source: Kidney Int. 2018 Apr;93(4):1000–7. doi: 10.1016/j.kint.2017.09.011 (PMC5978933; doi:10.1016/j.kint.2017.09.011)
Supplement: Figure S3 — Association between usual LDL cholesterol and the risk of (A) major vascular events, (B) vascular events of any type, (C) atherosclerotic vascular events, and (D) nonatherosclerotic vascular events. [file mmc5.pdf]

Supplementary Figure S3: Association between usual LDL−cholesterol and the risk of (A) major vascular events, (B) vascular events of any type, (C) atherosclerotic vascular events and (D) non−atherosclerotic vascular events

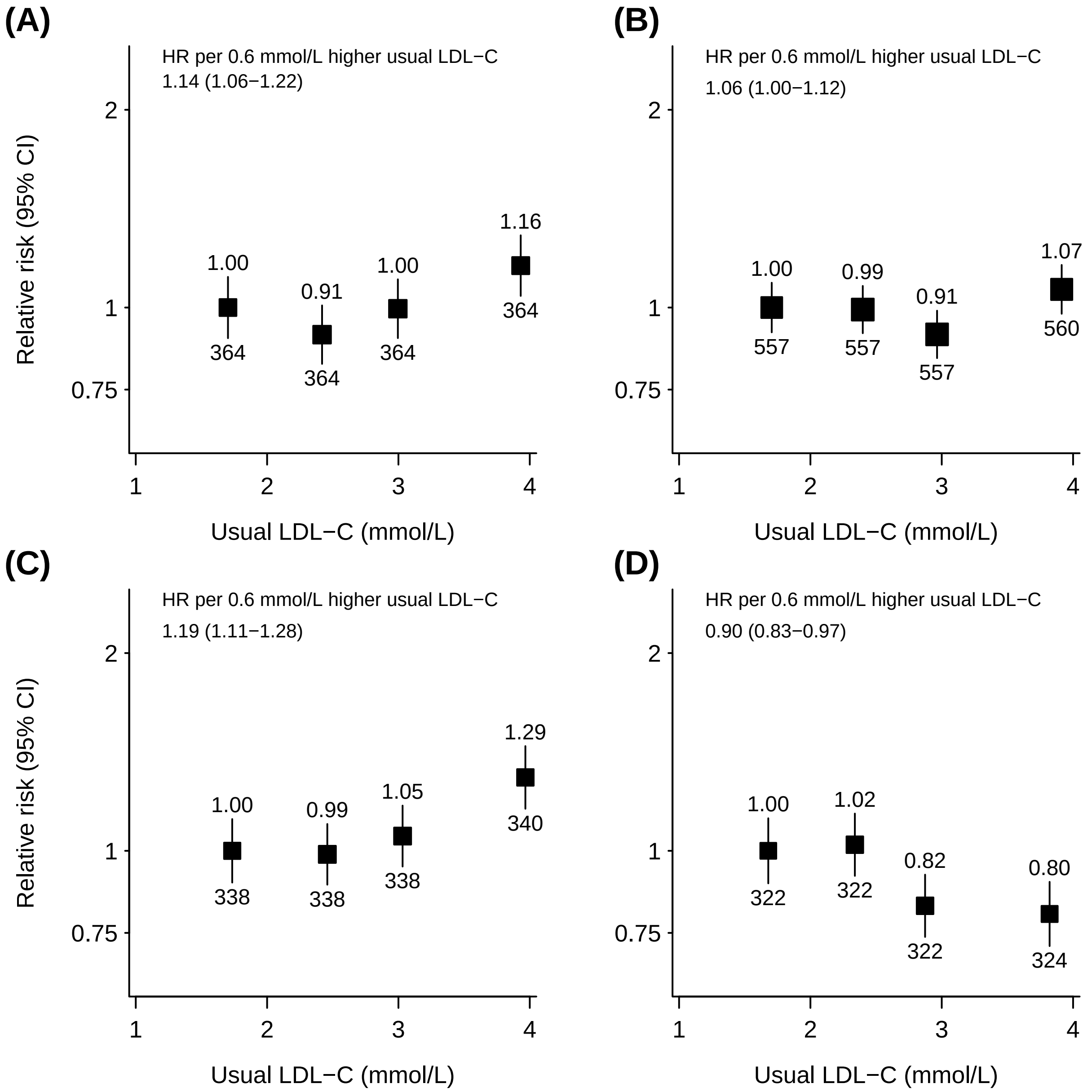

LDL−C=LDL cholesterol. See Supplementary Appendix for definitions of vascular events of any type, atherosclerotic vascular events and non−atherosclerotic vascular events. Hazard ratios adjusted for age, sex, ethnicity, treatment allocation, prior diabetes, prior vascular disease, smoking, BMI, HDL cholesterol and renal status are quoted (above squares) with numbers of events (below). Average HR (95% CI) throughout the range of values studied (i.e. assuming a log−linear relationship), corresponding to about a 1 SD difference in usual LDL−C.
