# Supplementary material for: Lowering LDL cholesterol reduces cardiovascular risk independently of presence of inflammation
Source: Kidney Int. 2018 Apr;93(4):1000–7. doi: 10.1016/j.kint.2017.09.011 (PMC5978933; doi:10.1016/j.kint.2017.09.011)

Supplementary figure S4: Effect of allocation to simvastatin plus ezetimibe on major vascular events, by level of C−reactive protein and dialysis status

	Δ LDL−C (mmol/L)	Simvastatin plus ezetimibe (n=4650)	Placebo (n=4620)	Risk ratio (95% CI)	p value
**Not on dialysis**					
**C−reactive protein (χ_1^2= 0.26, p=0.61)**					
<3.0	−0.95	179/1598 (11.2%)	232/1590 (14.6%)	0.76 (0.62−0.92)	
≥ 3.0 mg/L	−1.18	182/1300 (14.0%)	226/1331 (17.0%)	0.81 (0.67−0.99)	
**Subtotal: Not on dialysis**	**−1.06**	**390/3116 (12.5%)**	**494/3129 (15.8%)**	**0.78 (0.69−0.89)**	**0.0002**
**Dialysis**					
**C−reactive protein (χ_1^2= 0.51, p=0.48)**					
<3.0	−0.69	93/580 (16.0%)	87/530 (16.4%)	1.01 (0.76−1.36)	
≥ 3.0 mg/L	−0.90	195/842 (23.2%)	210/832 (25.2%)	0.89 (0.73−1.08)	
**Subtotal: Dialysis**	**−0.82**	**311/1534 (20.3%)**	**320/1491 (21.5%)**	**0.94 (0.80−1.09)**	**0.40**

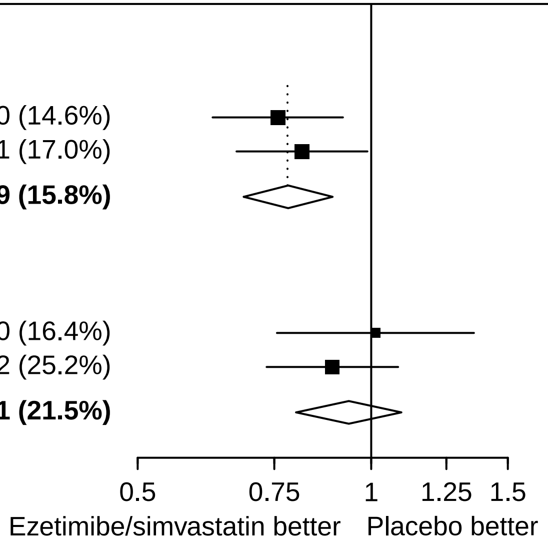

Supplement: Figure S4 — Effect of allocation to simvastatin plus ezetimibe on major vascular events, by level of C−reactive protein and dialysis status. [file mmc6.pdf]
