# Supplementary material for: Lowering LDL cholesterol reduces cardiovascular risk independently of presence of inflammation
Source: Kidney Int. 2018 Apr;93(4):1000–7. doi: 10.1016/j.kint.2017.09.011 (PMC5978933; doi:10.1016/j.kint.2017.09.011)
Supplement: Figure S6 — Association between LDL cholesterol and the risk of nonvascular events. [file mmc8.pdf]

Supplementary Figure S6: Association between LDL−cholesterol and the risk of non−vascular events

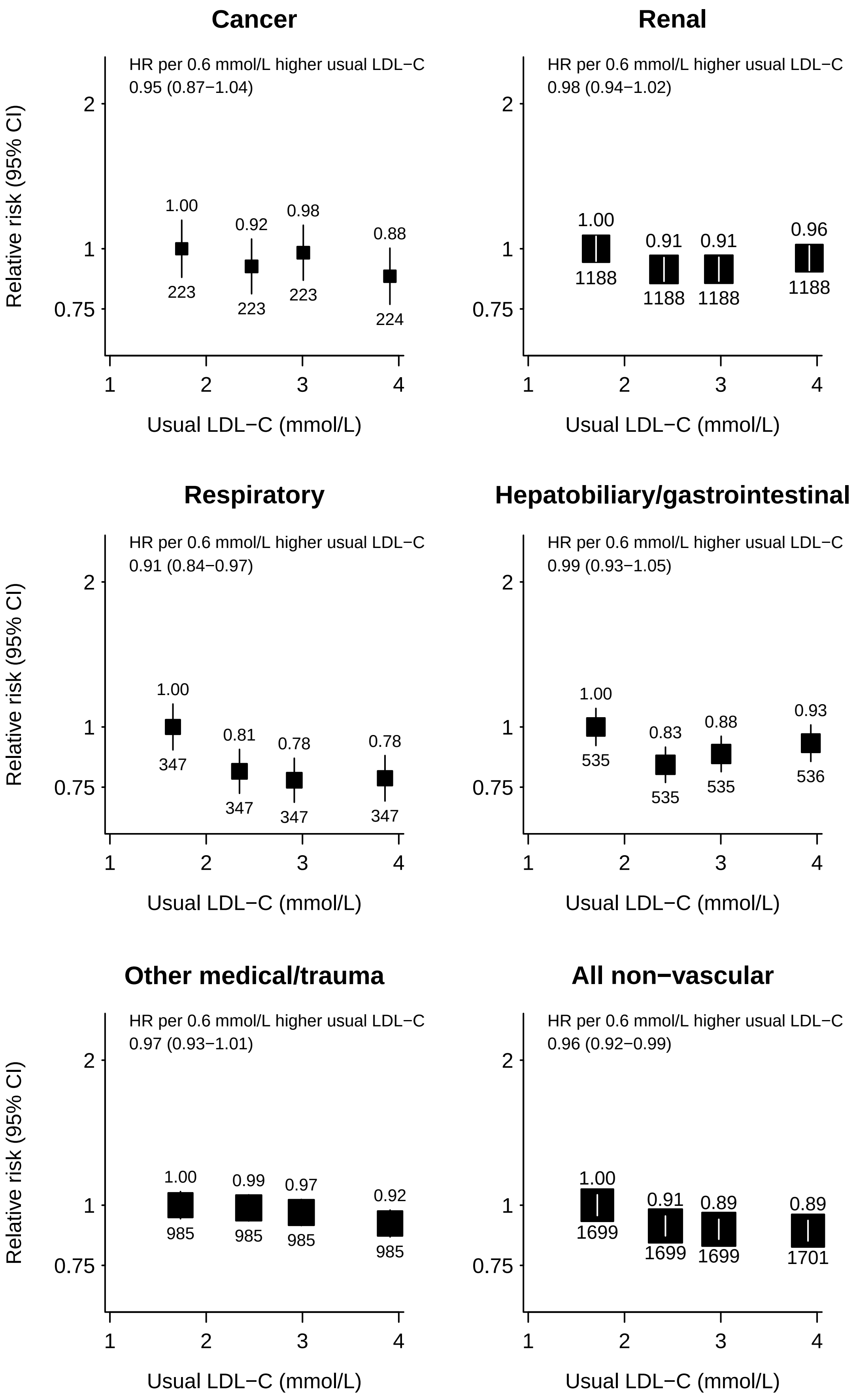

LDL−C=LDL−cholesterol. Hazard ratios adjusted for age, sex, ethnicity, treatment allocation, prior diabetes, prior vascular disease, smoking, BMI, HDL cholesterol and renal status are quoted (above squares) with numbers of events (below). Average HR (95% CI) throughout the range of values studied (i.e. assuming a log−linear relationship), corresponding to about a 1 SD difference in usual LDL−C.
